# Plotting receiver operating characteristic and precision–recall curves from presence and background data

**DOI:** 10.1002/ece3.7826

**Published:** 2021-07-01

**Authors:** Wenkai Li, Qinghua Guo

**Affiliations:** ^1^ Guangdong Provincial Engineering Research Center for Remote Sensing and Monitoring of Water Environment School of Geography and Planning Sun Yat‐Sen University Guangzhou China; ^2^ Institute of Ecology College of Urban and Environmental Sciences Peking University Beijing China

**Keywords:** area under the curve, model evaluation, precision–recall curve, presence and background data, receiver operating characteristic curve, species distribution modeling

## Abstract

1. The receiver operating characteristic (ROC) and precision–recall (PR) plots have been widely used to evaluate the performance of species distribution models. Plotting the ROC/PR curves requires a traditional test set with both presence and absence data (namely PA approach), but species absence data are usually not available in reality. Plotting the ROC/PR curves from presence‐only data while treating background data as pseudo absence data (namely PO approach) may provide misleading results.
2. In this study, we propose a new approach to calibrate the ROC/PR curves from presence and background data with user‐provided information on a constant *c*, namely PB approach. Here, *c* defines the probability that species occurrence is detected (labeled), and an estimate of *c* can also be derived from the PB‐based ROC/PR plots given that a model with good ability of discrimination is available. We used five virtual species and a real aerial photography to test the effectiveness of the proposed PB‐based ROC/PR plots. Different models (or classifiers) were trained from presence and background data with various sample sizes. The ROC/PR curves plotted by PA approach were used to benchmark the curves plotted by PO and PB approaches.
3. Experimental results show that the curves and areas under curves by PB approach are more similar to that by PA approach as compared with PO approach. The PB‐based ROC/PR plots also provide highly accurate estimations of *c* in our experiment.
4. We conclude that the proposed PB‐based ROC/PR plots can provide valuable complements to the existing model assessment methods, and they also provide an additional way to estimate the constant *c* (or species prevalence) from presence and background data.

## Graphical abstract

The receiver operating characteristic (ROC) and precision–recall (PR) plots have been widely used to evaluate the performances of species distribution models. We propose a new approach to calibrate the ROC/PR curves from presence and background data with user‐provided information on a constant *c*. An estimate of *c* can also be derived from the ROC/PR plots given that a model with good ability of discrimination is available.

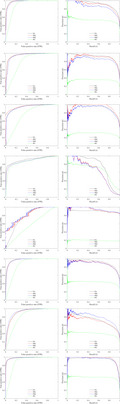

## 1 INTRODUCTION

Species distribution modeling (SDM) is an important tool to understand the statistical relationship between occurrences of species and environmental variables, and it has been applied in a variety of fields (Booth et al., 2014; Elith et al., 2006; Guisan & Thuiller, 2005; Peterson & Holt, 2003). For example, Kueppers et al. (2005) used discriminant analysis to study the potential ranges of two California endemic oaks in response to regional climate change. Hagar et al. (2020) used maximum entropy (MAXENT) to predict the habitat suitability of northern spotted owl in Oregon with forest structural attributes derived from airborne light detection and ranging data. When both observed presence and absence data are available, it is straightforward to apply standard binary classifiers such as logistic regression and neural network to predict the conditional probability of species occurrence at given locations (Guisan et al., 2002; Li et al., 2011; Marmion et al., 2009). However, reliable species absence data are usually not available in practice, which is referred to as the presence‐only problem (Elith et al., 2006). With presence‐only data, it is difficult to estimate the probability of species occurrence, so researchers usually estimate a relative index of habitat suitability instead (Elith et al., 2006; Hastie & Fithian, 2013; Phillips & Elith, 2013). One category of methods for presence‐only data is to train models using only presence data, such as ecological niche factor analysis (Hirzel et al., 2002), BIOCLIM (Booth, 2018; Busby, 1986), and DOMAIN (Carpenter et al., 1993). Another category of presence‐only methods involves generating pseudo absence or background data and combining them with observed presence data to train models, such as MAXENT, maximum likelihood analysis (MAXLIKE), inhomogeneous Poisson point process, naive logistic regression, and presence and background learning (Aarts et al., 2012; Keating & Cherry, 2004; Li et al., 2011; Phillips et al., 2006; Royle et al., 2012; Ward et al., 2009).

Model performance can be evaluated from two different aspects, namely calibration and discrimination (Jiménez‐Valverde et al., 2013; Lobo et al., 2008; Phillips & Elith, 2010). Calibration measures the agreement between predicted and true probabilities of species occurrence, whereas discrimination measures the ability to distinguish between presence and absence data (Phillips & Elith, 2010). In this study, we only focus on the aspect of discrimination. Using an independent test set consisting of both presence and absence data, we can generate a 2 × 2 confusion matrix to cross‐tabulate the binary predictions and observations, from which a variety of accuracy measures can be derived, such as overall accuracy, kappa statistic, true skill statistic (TSS), and *F*‐measure (Congalton, 1991; Fielding & Bell, 1997; Li & Guo, 2013; Liu et al., 2011). These accuracy measures consider both commission and omission errors, and they are threshold‐dependent, so a single threshold is required to convert the continuous outputs to binary outputs. Without absence data, however, commission error cannot be calculated, making model evaluation problematic with these traditional accuracy measures. To solve this problem, absolute validation index (AVI) and contrast validation index (CVI) were proposed to evaluate binary predictions without considering commission error (Hirzel et al., 2006). Li and Guo (2013) proposed two new statistics, namely *F*_cpb_ and *F*_pb_, to evaluate the predictive accuracy of binary predictions from presence and background data. *F*_cpb_ is an unbiased estimate of *F*‐measure, but it requires prior information of species prevalence. When species prevalence is not available, *F*_pb_ can be applied as a proxy of *F*‐measure, but it is only applicable to rank models for the same species because its upper bound is affected by the unknown prevalence. Liu et al. (2013) proved that maximizing TSS from presence and pseudo absence data is equivalent to maximizing TSS from presence and absence data in terms of threshold selection.

The receiver operating characteristic (ROC) curve and area under the ROC curve (AUCROC) have also been commonly used for model evaluation in SDM (Fielding & Bell, 1997). Unlike the threshold‐dependent measures that rely on a single threshold, the ROC curve and AUCROC evaluate model performance by considering all possible thresholds, so they are applicable to the continuous outputs without requiring thresholding. Alternatively, users can plot the precision–recall (PR) curve and calculate area under the PR curve (AUCPR) to evaluate model performance (Davis & Goadrich, 2006). Please note that the ROC curve incorporates correctly predicted absence sites (true negative), and hence, AUCROC value is influenced by total geographic extent (Lobo et al., 2008). When species prevalence is very small or the geographic extent is very large, AUCROC value may be inflated unrealistically (Sofaer et al., 2019). By contrast, the PR curve ignores true negative, so it is more robust to geographic extent and suitable for species with small prevalence (Leroy et al., 2018; Sofaer et al., 2019). In other words, the ROC curve is more suitable for balanced datasets whereas the PR curve is more suitable for imbalanced datasets (Davis & Goadrich, 2006; Saito & Rehmsmeier, 2015; Sofaer et al., 2019).

Essentially, the ROC and PR curves are based on both commission and omission errors, so they also suffer from the presence‐only problem. Currently, it is a common practice to plot the ROC/PR curves and calculate area under the curve (AUC) by treating the background data as absence data in the literature, but researchers have pointed out that this approach can make the results misleading and difficult to interpret because background data are actually contaminated by presence data (Jiménez‐Valverde, 2012; Peterson et al., 2008; Phillips et al., 2006). Li and Guo (2013) have proved that both recall (inversely related to omission error) and precision (inversely related to commission error) can be unbiasedly estimated from presence and background data given that species prevalence is available, thus making it possible to plot the correct ROC/PR curves without absence data. However, this approach has not yet been applied to correct the ROC/PR curves from presence and background data in the field of SDM. In this study, therefore, we aim to investigate the following two questions. Given true species prevalence, can we plot the correct ROC/PR curves from presence and background data? Without true species prevalence, can we estimate prevalence from presence and background data?

## 2 MATERIALS AND METHODS

### 2.1 Model evaluation with a nontraditional test set

An independent test set with random samples drawn from the population is required for model evaluation. Let *y* = 1 denote presence data and *y* = 0 denote absence data; *s* = 1 denote labeled data and *s* = 0 denote unlabeled data. A traditional test set contains fully labeled presence–absence data randomly sampled from the population. A nontraditional test set contains labeled and unlabeled data, in which only presence data are labeled and unlabeled data are a mixture of presence and absence data whose labels are unknown. In other words, the labeled data (*s* = 1) must be presence data (*y* = 1), but unlabeled data (*s* = 0) may be presence (*y* = 1) or absence (*y* = 0) data in a nontraditional test set. By comparing the true labels and binary predictions on a traditional test set, we can generate a confusion matrix with four quadrants: true positive (*TP*), false positive (*FP*), false negative (*FN*), and true negative (*TN*). If we simply treat the unlabeled data as absence data in a nontraditional test set, we also create a confusion matrix with the four quadrants denoted differently: true positive (*TP*′), false positive (*FP*′), false negative (*FN*′), and true negative (*TN*′) (see Table 1).

**TABLE 1:** Confusion matrices from traditional and nontraditional test sets

Prediction	Reference			
	Traditional test set		Nontraditional test set	
	*y* = 1	*y* = 0	*s* = 1	*s* = 0
*y*′ = 1	*TP*	*FP*	*TP*′	*FP*′
*y*′ = 0	*FN*	*TN*	*FN*′	*TN*′

From a traditional confusion matrix, we can calculate precision (*p*), recall (*r*), and false‐positive rate (*FPR*) using the following equations:

(1) $$p=\frac{\mathrm{TP}}{TP+FP}$$

(2) $$r=\frac{\mathrm{TP}}{TP+FN}$$

(3) $$FPR=\frac{\mathrm{FP}}{FP+TN}$$

The species prevalence *P*(*y* = 1) and the proportion of predicted presences *P*(*y*′ = 1) can be calculated through the following equations:

(4) $$P\left(y=1\right)=\frac{TP+FN}{TP+FN+FP+TN}$$

(5) $$P\left(y^{\prime }=1\right)=\frac{TP+FP}{TP+FN+FP+TN}$$

Therefore, we can rewrite Equation (3) as:

(6) $$FPR=\frac{\mathrm{FP}}{FP+TN}=\frac{P\left(y^{\prime }=1\right)\times \left(1‐p\right)}{1‐P\left(y=1\right)}$$

**FIGURE 1:**
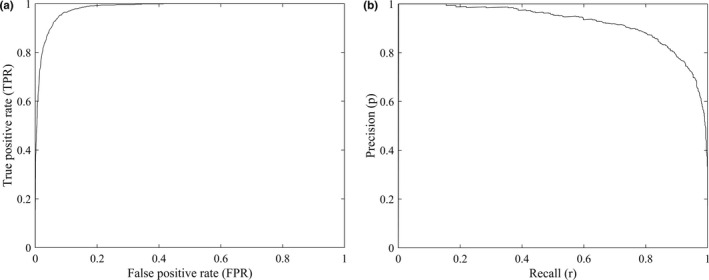
Examples of the ROC (a) and PR (b) curves

By considering all possible thresholds, the ROC curve plots true‐positive rate (*TPR*) versus *FPR*, whereas the PR curve plots *p* versus *r* (Figure 1). *TPR* is exactly the same as *r* that is related omission error (*FN*), and both *FPR* and *p* are related to commission error (*FP*), so we can connect the ROC and PR curves through Equation (6). As the discrimination ability of a model increases, the curves in Figure 1 will shift upward, that is, the ROC curve will shift toward the point (0, 1) whereas the PR curve will shift toward the point (1, 1).

On a nontraditional test set, only a proportion of presence data are labeled and the labels of absence data are unknown, so the traditional confusion matrix cannot be completely determined. Here, we consider two common sampling scenarios: single‐training‐set (Elkan & Noto, 2008) and case–control (Lancaster & Imbens, 1996). In the single‐training‐set scenario, we visit a number of sites randomly distributed within the study area, and a site is labeled as presence if species occurrence is observed or unlabeled otherwise. In the case–control scenario, the labeled presence data are randomly sampled from the presence subset, and unlabeled data are randomly sampled from the population. Let *c* = *P*(*s* = 1|*y* = 1) define the probability that species occurrence is detected (labeled), that is, the ratio of labeled presence data to the total number of presence data in a test set (Li et al., 2011). The value of *c* is usually smaller than one, so unlabeled data actually contain both presence and absence data. Because species absence is difficult to observe, the presence–absence survey data can also be interpreted as presence‐unlabeled data in the single‐training‐set scenario, whereas the presence‐background data can be interpreted as presence‐unlabeled data in the case–control scenario.

With a nontraditional test set, we can define the following measures:

(7) $$p^{\prime }=\frac{Tp^{\prime }}{Tp^{\prime }+Fp^{\prime }}$$

(8) $$r^{\prime }=\frac{Tp^{\prime }}{Tp^{\prime }+FN^{\prime }}$$

Because *r*′ is calculated from the observed (labeled) presence data, we have *r*′ = *r*. However, *p*′ is not equal to *p* because it is calculated from unlabeled data. According to Li and Guo (2013), *p*′ and *p* have the following relationship:

(9) $$p=\frac{1‐c}{c}\times \frac{p^{\prime }}{1‐p^{\prime }}$$

in the case–control scenario. In the single‐training‐set scenario, their relationship is slightly different, which should be:

(10) $$p=p^{\prime }/c$$

Here, we use Table 2 to illustrate the derivations of Equations (9) and (10). In Table 2, *m*_1_ and *m*_4_ can be calculated from labeled data, but *m*_2_, *m*_3_, *m*_5_, and *m*_6_ cannot be calculated because the true labels of unlabeled data are not known. Meanwhile, the total number of labeled data *n*_1_, the total number of unlabeled data *n*_0_, the total number of predicted presences *k*_1_, the total number of predicted absences *k*_0_, and the total number of test data *t* are known. According to the definitions of *c*, *p*′ and *r*′, we have the following equations:

(11) $$c=\frac{m_{1}+m_{4}}{m_{1}+m_{2}+m_{4}+m_{5}}$$

(12) $$p^{\prime }=\frac{m_{1}}{m_{1}+m_{2}+m_{3}}$$

(13) $$r^{\prime }=\frac{m_{1}}{m_{1}+m_{4}}$$

**TABLE 2:** A confusion matrix from a nontraditional test set

Prediction	Reference			Total
	*s* = 1	*s* = 0		
	*y* = 1	*y* = 1	*y* = 0	
*y*′ = 1	*m*_1_	*m*_2_	*m*_3_	*k*_1_ = *m*_1_ + *m*_2_ + *m*_3_
*y*′ = 0	*m*_4_	*m*_5_	*m*_6_	*k*_0_ = *m*_4_ + *m*_5_ + *m*_6_
Total	*n*_1_ = *m*_1_ + *m*_4_	*n*_0_ = *m*_2_ + *m*_3_ + *m*_5_ + *m*_6_		*t* = *n*_1_ + *n*_0_

Note:

Numbers with shade are known, and numbers without shade are not known.

In the case–control scenario, the unlabeled data are randomly sampled from the population, so *p*, *r*, *P*(*y* = 1), and *P*(*y*′ = 1) can be calculated as:

(14) $$p=\frac{m_{2}}{m_{2}+m_{3}}$$

(15) $$r=\frac{m_{2}}{m_{2}+m_{5}}$$

(16) $$P\left(y=1\right)=\left(m_{2}+m_{5}\right)/n_{0}$$

(17) $$P\left(y^{\prime }=1\right)=\left(k_{1}‐m_{1}\right)/n_{0}$$

According to Equations (11) and (12), we have:

(18) $$\frac{1‐c}{c}\times \frac{p^{\prime }}{1‐p^{\prime }}=\frac{m_{2}+m_{5}}{m_{1}+m_{4}}\times \frac{m_{1}}{m_{2}+m_{3}}=\frac{m_{1}}{m_{1}+m_{4}}\times \frac{m_{2}+m_{5}}{m_{2}+m_{3}}$$

Because *r* = *r*′, substituting Equations (13), (14), (15) to Equation (18), we have:

(19) $$\frac{1‐c}{c}\times \frac{p^{\prime }}{1‐p^{\prime }}=\frac{m_{2}}{m_{2}+m_{5}}\times \frac{m_{2}+m_{5}}{m_{2}+m_{3}}=p$$

which proves the relationship between *p* and *p*′ in Equation (9) under the case–control scenario. Please note that (1 − *c*)/*c* here is equal to the reciprocal of the constant term *c* in Li and Guo (2013), so Equation (9) of this article is equivalent to Equation (9) in Li and Guo (2013).

Unlike the case–control scenario where unlabeled data alone are random samples of the population, the combined labeled and unlabeled data together constitute random samples of the population in the single‐training‐set scenario, so *p*, *r*, *P*(*y* = 1), and *P*(*y*′ = 1) are calculated differently:

(20) $$p=\frac{m_{1}+m_{2}}{m_{1}+m_{2}+m_{3}}$$

(21) $$r=\frac{m_{1}+m_{2}}{m_{1}+m_{2}+m_{4}+m_{5}}$$

(22) $$P\left(y=1\right)=\left(m_{1}+m_{2}+m_{4}+m_{5}\right)/t$$

(23) $$P\left(y^{\prime }=1\right)=k_{1}/t$$

According to Equations (11) and (12), we have:

(24) $$\frac{p^{\prime }}{c}=\frac{m_{1}}{m_{1}+m_{2}+m_{3}}\times \frac{m_{1}+m_{2}+m_{4}+m_{5}}{m_{1}+m_{4}}=\frac{m_{1}}{m_{1}+m_{4}}\times \frac{m_{1}+m_{2}+m_{4}+m_{5}}{m_{1}+m_{2}+m_{3}}$$

Since *r* = *r*′, substituting Equations (13), (20), (21), (22), (23), (24), we have:

(25) $$\frac{p^{\prime }}{c}=\frac{m_{1}+m_{2}}{m_{1}+m_{2}+m_{4}+m_{5}}\times \frac{m_{1}+m_{2}+m_{4}+m_{5}}{m_{1}+m_{2}+m_{3}}=p$$

which proves the relationship between *p* and *p*′ in Equation (10) under the single‐training‐set scenario.

Please note that *c* has the same definition in both scenarios, that is, *c* = *P*(*s* = 1|*y* = 1), but its relationship with species prevalence is different in two scenarios:

(26) $$c=n_{1}/\;\left[n_{1}+n_{0}\times P\left(y=1\right)\right]$$

according to Equations (11) and (16) in the case–control scenario;

(27) $$c=\;n_{1}/\left[t\times P\left(y=1\right)\right]$$

according to Equations (11) and (22) in the single‐training‐set scenario. Given a nontraditional test set, *c* is a fixed constant whose value is affected by the number of labeled data (*n*_1_), the number of unlabeled data (*n*_0_), and species prevalence. Meanwhile, *P*(*y*′ = 1) is equal to the proportion of predicted presences among the unlabeled set in the case–control scenario, or equal to the proportion of predicted presences among the whole test set in the single‐training‐set scenario. According to Equation (6), *FPR* can be determined if *p*, *P*(*y*′ = 1), and *P*(*y* = 1) are known. Therefore, if species prevalence is available, we can calculate *p*, *r*, and *FPR* from a nontraditional test set, and then plot the corrected ROC/PR curves.

Here, the key information is the species prevalence or the constant *c*. If one of them is known, the other one can be determined as well. In real‐world applications, however, species prevalence and hence the constant *c* are usually unknown. Although species prevalence is normally unidentifiable without absence data, it can be estimated under certain assumptions or conditions (Hastie & Fithian, 2013; Lancaster & Imbens, 1996; Li et al., 2011; Phillips & Elith, 2013; Royle et al., 2012; Ward et al., 2009). Here, we propose to estimate *c* from the ROC/PR curves under the condition that a model with good discrimination ability exists. When we increase the threshold to produce binary predictions, the omission error will increase but the commission error will decrease. If a model has a good ability to separate presence from absence data, we can set a high threshold to minimize the commission error, so *p* will reach its maximum value of one and *FPR* will reach its minimum value of zero. The ROC/PR curves in Figure 1 are produced by a model that satisfies the condition of good discrimination ability, from which we can observe that the ROC curve passes the positions with minimum *FPR* in the lower left corner and the PR curve passes the positions with maximum *p* in the upper left corner.

According to Equations (9) and (10), *p* is a monotonically increasing function of *p*′, so we can find the maximum value of *p*′ (i.e., the highest point in the PR curve or the most left point in the ROC curve) to infer the constant *c*. Because the maximum value of *p*′ (denoted as *p*′_max_) is the position where *p* = 1, we obtain *c* = *p*′_max_ according to Equation (9) or (10). However, estimating *c* using a single point in the ROC/PR curve may result in a large variance, so we propose to select multiple points whose values of *p*′ are relatively high to estimate *c*. Let *PP* be a subset of points in the ROC/PR curve whose values of *p*′ fall within a range of user‐specified percentiles. We have the following estimator:

(28) $$c=\frac{1}{j}\sum_{i\in PP}p_{i}^{\prime }$$

where *j* is the cardinality of *PP*. For example, we can select those points where *p*′ falls between 90th and 99th percentiles across all possible thresholds. Once *c* is estimated, species prevalence can be estimated as well according to Equation (26) or (27).

### 2.2 Experimental design

In this section, we investigate the effectiveness of the proposed method to correct the ROC/PR curves from presence and background data, which is the case–control scenario commonly used in SDM. We trained different models from presence and background data, and model performances were evaluated using a traditional test set with presence–absence data and a nontraditional test set with presence‐background data, respectively. The ROC/PR curves were plotted using three different approaches: standard presence–absence (PA) approach, presence‐only (PO) approach by simply treating background data as absence data, and presence‐background (PB) approach using the proposed method to calibrate the curves. The curves produced by PO and PB approaches were compared with the benchmark curves produced by PA approach. Because it is difficult to obtain reliable species absence data in reality, we used virtual species in our experiment, which has become a common approach to test models from different aspects (Duan et al., 2015; Hirzel et al., 2001; Li et al., 2011; Meynard & Kaplan, 2013). One‐class classification of remote sensing imagery is similar to SDM in that the same models and the same accuracy measures can be applied in both fields, and it is possible to collect reliable absence data in image classification, so we also used a real aerial photograph to test the proposed method.

### 2.3 Dataset

We simulated five virtual species with different prevalence values following the procedure of Li et al. (2011). The conditional probability of species occurrence *P*(*y* = 1|*x*) was modeled using the logistic transform of a linear function defined in Equation (29) or a quadratic function defined in Equation (30):

(29) $$P\left(y=1|x\right)=\frac{e^{b_{0}+b_{1}x_{1}+b_{2}x_{2}+b_{3}x_{3}}}{1+e^{b_{0}+b_{1}x_{1}+b_{2}x_{2}+b_{3}x_{3}}}$$

(30) $$P\left(y=1|x\right)=\frac{e^{b_{0}+b_{1}{\left(x_{1}‐{\bar{x}}_{1}\right)}^{2}+b_{2}{\left(x_{2}‐{\bar{x}}_{2}\right)}^{2}+b_{3}{\left(x_{3}‐{\bar{x}}_{3}\right)}^{2}}}{1+e^{b_{0}+b_{1}{\left(x_{1}‐{\bar{x}}_{1}\right)}^{2}+b_{2}{\left(x_{2}‐{\bar{x}}_{2}\right)}^{2}+b_{3}{\left(x_{3}‐{\bar{x}}_{3}\right)}^{2}}}$$

where *b*_i_ is a coefficient (see Table 3) and *x*_i_ is an environmental variable; ${\bar{x}}_{i}$ is the mean of *x*_i_. We considered three environmental variables in California with an extent of 410,003 km^2^, including annually average precipitation, annually average temperature, and elevation, all of which were extracted from the WorldClim database (https://worldclim.org/) with a spatial resolution of 1 km (Fick & Hijmans, 2017). At each pixel, we used a random number (0 <= *q* < 1) to generate realized binary labels, that is, presence (*y* = 1) if *q* < *P*(*y* = 1|*x*) or absence (*y* = 0) if *q* >= *P*(*y* = 1|*x*). From the realized binary map, we drew a nontraditional training set and a nontraditional test set, separately, both of which contained case–control presence‐background data. The number of presence data in the test set was 1,000, whereas the number of presence data in the training set varied, including 10, 50, 100, 500, and 1,000. The number of background data in the training/test set was five times of presence data. With virtual species, we actually know the true labels of random background data, so we also used them to constitute a traditional test set. The training and test sets were randomly realized ten different times, and the experimental results were averaged in our analysis.

**TABLE 3:** Prevalence and coefficients of five virtual species

Species	Prevalence	Coefficients				Function
		*b*_0_	*b*_1_	*b*_2_	*b*_3_	
Spec1	0.1638	−10	−0.15	−2.5	28	Linear
Spec2	0.3298	0.5	−1.5	−8.5	18	Linear
Spec3	0.4471	−0.4	−0.8	−5	18	Linear
Spec4	0.0503	−5	−0.2	5	30	Quadratic
Spec5	0.7837	2.8	5	−5	−50	Quadratic

The aerial photograph in Li et al. (2021) was also used to test the proposed method. The image covers an extent of 500 m × 500 m in the city of El Cerrito in California, with a spatial resolution of 0.3 m. The total number of pixels is 2,778,889, and the prevalence values of urban, tree, and grass are 0.2292, 0.2106, and 0.1880, respectively. We performed different one‐class classifications to map different land types (i.e., urban, tree, and grass), separately, treating them as three different species. For each land type, we drew a nontraditional training set and a nontraditional test set, respectively, following the case–control sampling scheme. The number of presence data in the test set was 2,000, and the number of presence data in the training set was set as 200, 1,000, and 5,000, respectively. The number of background data was five times of presence data in both training and test sets. Again, the true labels of background data in the nontraditional test set can be obtained through manual interpretation, so we also used these background data to constitute a traditional test set. Both the training and test sets were randomly realized ten different times, and the experimental results were averaged in our analysis.

We trained different classifiers using different sample sizes to produce different model performances. For convenience, we refer to sample size as the number of labeled presence data in a training set throughout this paper. We selected both simple and complicated classifiers, but the purpose here was only to produce poor and good predictions, and it does not matter what specific methods were used. For the virtual species, we trained DOMAIN (Carpenter et al., 1993), generalized linear model (GLM) (Guisan et al., 2002), and artificial neural network (ANN) (Hecht‐Nielsen, 1989) using five sample sizes (i.e., 10, 50, 100, 500, and 1,000); for the aerial photograph, we trained GLM and ANN using three sample sizes (i.e., 200, 1,000, and 5,000) and convolutional neural network (CNN) (Lecun et al., 1998) with only one sample size (i.e., 5,000). DOMAIN was trained from only presence data whereas other classifiers were trained from presence‐background data. All of the models were evaluated by a traditional test set and a nontraditional test set, respectively. We plotted the ROC/PR curves and calculated AUC values using PA, PO, and PB approaches. For the PB approach, we tested two different scenarios: true species prevalence was given (denoted as PB1) and species prevalence was estimated from the ROC/PR curves (denoted as PB2). In real‐world applications, PB1 could be applied when there is independent presence–absence survey data or expert knowledge to provide information on prevalence, whereas PB2 could be applied when there is no prior information on prevalence.

## 3 RESULTS

**FIGURE 2:**
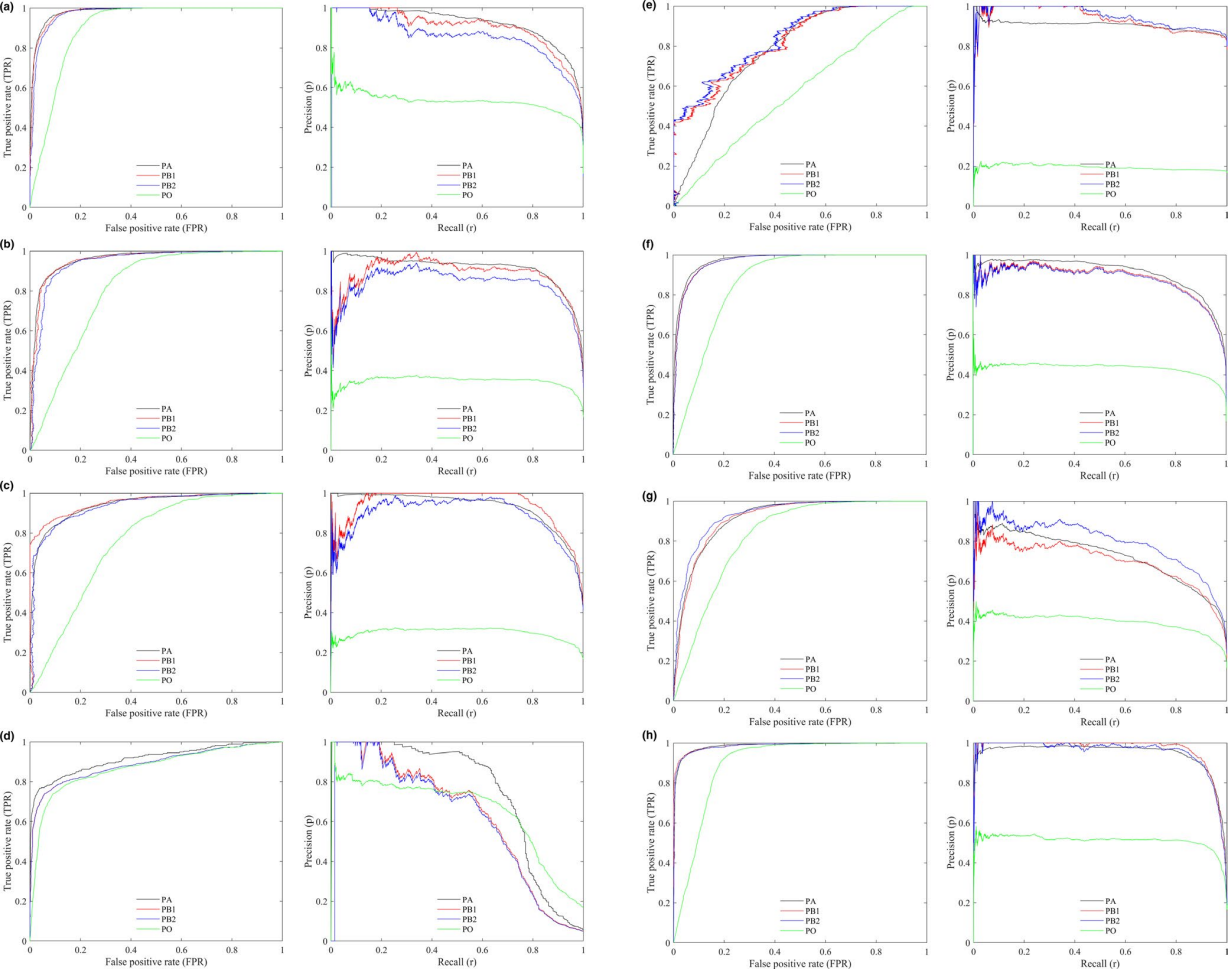
The ROC (left) and PR (right) curves by PA, PB, and PO approaches. PB1: prevalence is given; PB2: prevalence is estimated. Model: ANN trained with a sample size of 1,000. Virtual species: spec1 (a); spec2 (b); spec3 (c); spec4 (d); spec5 (e). Aerial photograph: urban (f); tree (g); grass (h)

**FIGURE 3:**
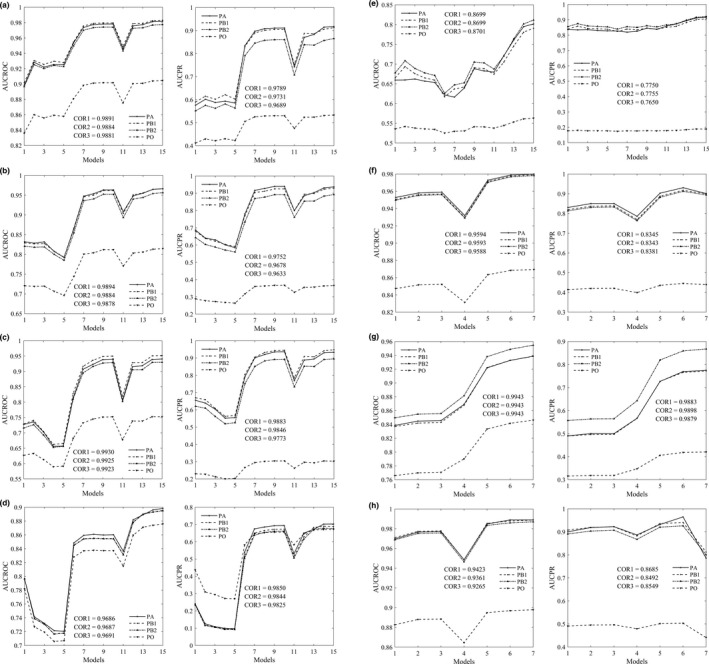
The average values of AUCROC (left) and AUCPR (right) over ten random realizations by PA, PB, and PO approaches. PB1: prevalence is given; PB2: prevalence is estimated. Virtual species: spec1 (a); spec2 (b); spec3 (c); spec4 (d); spec5 (e). Aerial photograph: urban (f); tree (g); grass (h). For the virtual species, models 1–15 refer to DOMAIN with five sample sizes, GLM with five samples, and ANN with five samples, respectively. For the aerial photograph, models 1–7 refer to GLM with three sample sizes, ANN with three sample sizes, and CNN with one sample size, respectively. COR1: the correlation coefficient between PA and PB1. COR2: the correlation coefficient between PA and PB2. COR3: the correlation coefficient between PA and PO

In Figure 2, we present part of the ROC/PR curves by different approaches. Generally, the curves produced by PB approach are quite similar to the benchmark curves by PA approach, whereas the curves produced by PO approach are obviously lower than that by PA approach for all species. Meanwhile, the discrepancies between PR curves are obviously larger than that between ROC curves. According to Figure 3, the rankings of models by AUC values are similar for PA, PB, and PO approaches, and the correlations of AUC values between different approaches are strong in general. The correlation coefficient of AUC between PA and PB is slightly higher than that between PA and PO approaches. For example, the correlation coefficient of AUCPR between PA and PB is 0.9789 when true prevalence is given or 0.9731 when prevalence is estimated, whereas the correlation coefficient of AUCPR between PA and PO is 0.9689 for the virtual species spec1.

Based on the ranking of models by PO approach, we used the ROC (or PR) curve produced by the best model with the highest AUCROC (or AUCPR) value to estimate the constant *c* and prevalence, which are shown in Table 4. As can be seen, the accuracies of estimated prevalence and *c* are relatively high in most cases. For example, the true values of prevalence and *c* for urban are 0.2292 and 0.4660, respectively, and the estimated values are 0.2275 and 0.4678, respectively. For the virtual species, the absolute value of relative error of prevalence ranges from 2% to 8%, whereas the absolute value of relative error of *c* ranges from 1% to 6%. For the aerial photograph, the absolute value of relative error of prevalence ranges from 0% to 14%, whereas the absolute value of relative error of *c* ranges from 0% to 7%. The highest accuracy is produced by classification of urban, and the largest error is produced by classification of tree from the real aerial photograph.

**TABLE 4:** The true and estimated values of prevalence and *c*

Type	True value		Estimated value		Relative error (%)	
	*P*(*y* = 1|*x*)	*c*	*P*(*y* = 1|*x*)	*c*	*P*(*y* = 1|*x*)	*c*
Spec1	0.1638	0.5498	0.1531	0.5664	−6.53	3.02
Spec2	0.3298	0.3775	0.3117	0.3908	−5.48	3.54
Spec3	0.4471	0.3091	0.4128	0.3263	−7.66	5.59
Spec4	0.0503	0.7991	0.0490	0.8032	−2.60	0.52
Spec5	0.7837	0.2033	0.7988	0.2002	1.93	−1.51
Urban	0.2292	0.4660	0.2275	0.4678	−0.74	0.39
Tree	0.2106	0.4871	0.2394	0.4552	13.68	−6.56
Grass	0.1880	0.5154	0.1839	0.5210	−2.18	1.08

**FIGURE 4:**
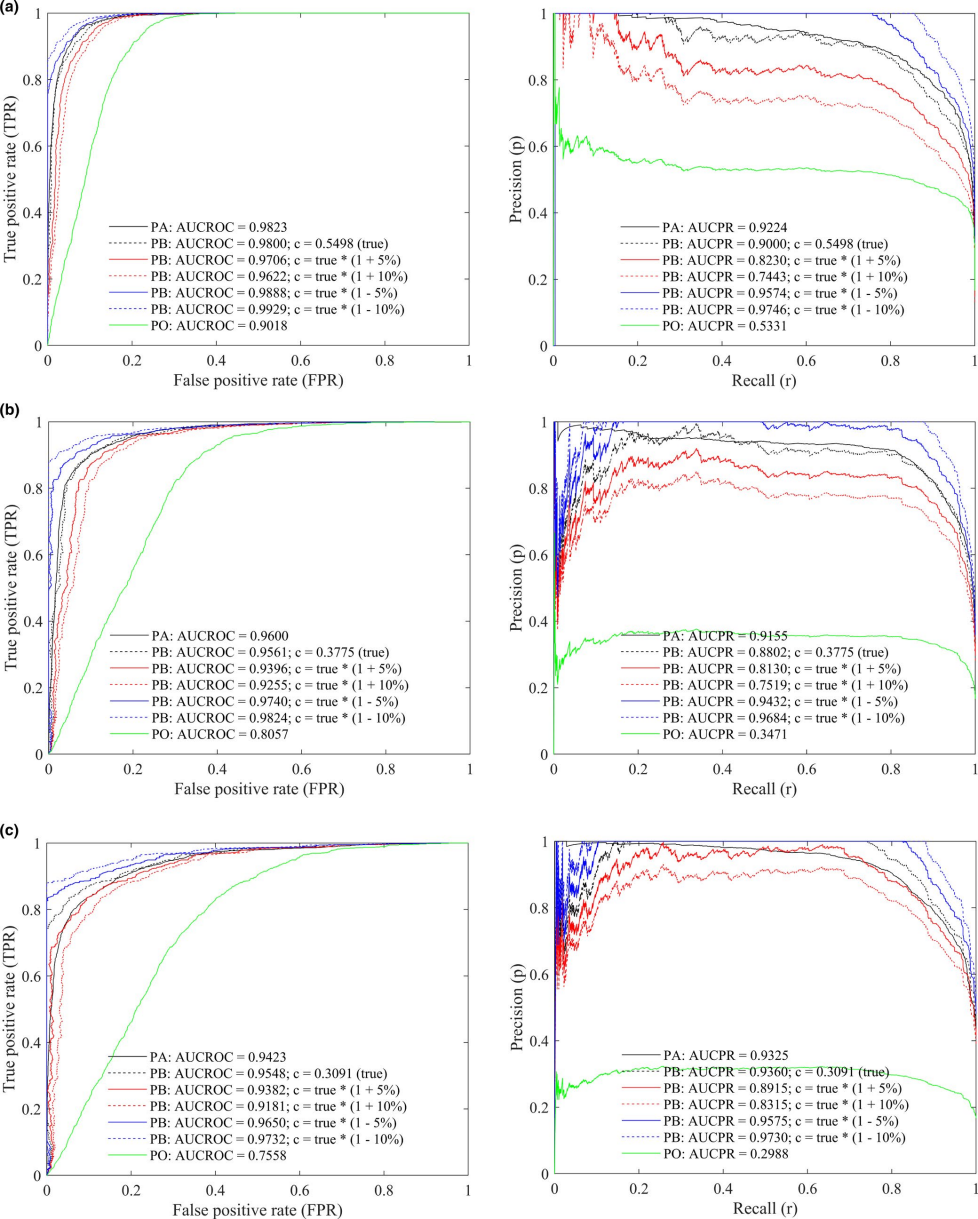
The sensitivity of ROC (left) and PR (right) curves to constant *c* by PB approach for the virtual species: spec1 (a); spec2 (b); spec3 (c). Model: ANN trained with a sample size of 1,000

According to Table 4, the largest absolute value of relative error of estimated *c* in our experiment is 6.56%. The sensitivity of the calibrated curves by PB to the constant *c* is shown in Figure 4, in which the ROC/PR curves are plotted using the true value of *c* with additive relative errors of ±10%. We can see that the ROC curve moves rightward and the PR curve moves downward when *c* is overestimated, and this trend switches to the opposite direction correspondently when *c* is underestimated. Consequently, the AUCROC and AUCPR values are underestimated when *c* is overestimated, whereas they are overestimated when *c* is underestimated. Take the virtual species spec1 as an example, the values of AUCROC and AUCPR for PA approach are 0.9823 and 0.9224, respectively; the values of AUCROC and AUCPR for PB approach are 0.9800 and 0.9000, respectively, when the true value of *c* is given; the values of AUCROC and AUCPR for PB approach are 0.9622 and 0.7443, respectively, when *c* with +10% relative error is given; the values of AUCROC and AUCPR for PB approach are 0.9929 and 0.9746, respectively, when *c* with −10% relative error is given. Meanwhile, the variation of PR curve by PB approach with different values of *c* is larger than the variation of ROC curve.

**FIGURE 5:**
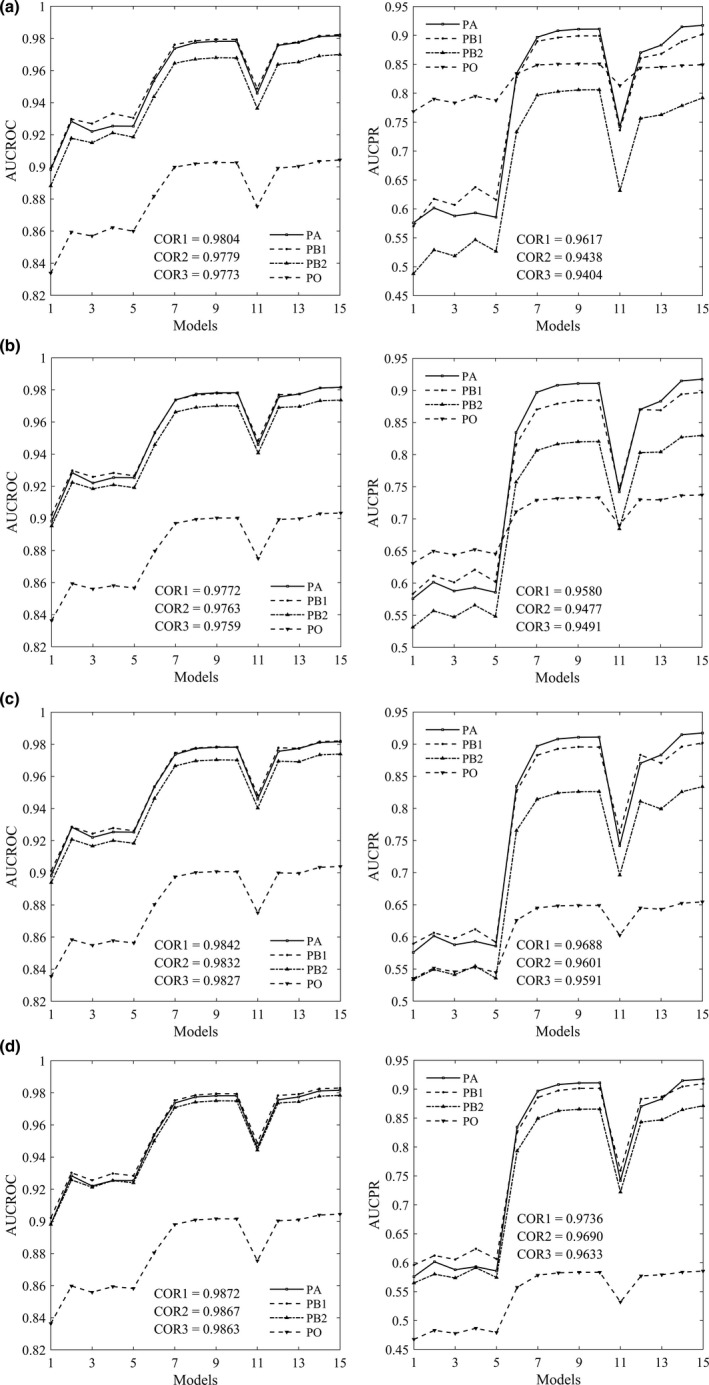
The average values of AUCROC (left) and AUCPR (right) over ten random realizations by PA, PB, and PO approaches for the virtual species spec1. The number of presence data in the test set is fixed as 1,000, and the number of background data is set as 1,000 (a), 2,000 (b), 3,000 (c), and 4,000 (d). PB1: prevalence is given; PB2: prevalence is estimated. Models 1–15 refer to DOMAIN with five sample sizes, GLM with five samples, and ANN with five samples, respectively. COR1: the correlation coefficient between PA and PB1. COR2: the correlation coefficient between PA and PB2. COR3: the correlation coefficient between PA and PO

The effects of the ratio of presence to background data in the test set are shown in Figure 5. With different ratio values, the AUC values produced by PB approach are close to that produced by PA approach consistently, and the curves by PA and PB approaches are almost unaffected by the ratio. Similarly, the AUCROC values by PO approach are almost the same with different ratio values. However, the AUCPR curve by PO approach is greatly affected by the ratio value. For example, as the ratio changes from 1:1 to 1:4, the range of AUCPR curve by PO approach changes from [0.77, 0.85] to [0.47 0.59].

The estimates of *c* by models with different abilities of discrimination are shown in Table 5. By switching the true probability values between *P*(*y* = 1|*x*) and *P*(*y* = 0|*x*) at different proportions (i.e., 40%, 30%, 20%, and 10%) of randomly selected pixels, we obtained different synthetic models of different discrimination abilities for the virtual species spce1, with AUCROC values ranging from 0.6 to 0.9 and AUCPR values ranging from 0.2 to 0.5. Overall, the estimated values of *c* by the synthetic models with different levels of AUC are accurate relatively. For the synthetic model with AUCROC of 0.5955, the estimated value of *c* is 0.5830 whereas the true value of *c* is 0.5498, with a relative error of 6.04%.

**TABLE 5:** The true and estimated values of *c* for the virtual species spec1 by synthetic models with different AUC values

Model	AUCROC	AUCPR	True value	Estimated value	Relative error (%)
1	0.5955	0.1899	0.5498	0.5830	6.04
2	0.6928	0.2407	0.5498	0.5672	3.17
3	0.7906	0.3211	0.5498	0.5671	3.15
4	0.8844	0.4675	0.5498	0.5565	1.22

Note

The synthetic models were produced by switching the true probability values between *P*(*y* = 1|*x*) and *P*(*y* = 0|*x*) at a number of randomly selected pixels.

## 4 DISCUSSION

Developing novel methods to evaluate the performance of models without absence data is important in SDM since reliable absence data are usually not available in real‐world applications. Currently, one of the most commonly used approaches to address the presence‐only problem in SDM is to train models using presence and background data, which belongs to the case–control sampling scenario, and models are usually evaluated using the ROC/PR plots by simply treating the background data as absence data (Jiménez‐Valverde, 2012; Lobo et al., 2008; Peterson et al., 2008; Phillips et al., 2006; Sofaer et al., 2019). This PO approach can rank the models by the relative value of AUC, but the absolute value of AUC may be quite different from its true value and hence is misleading (Lobo et al., 2008; Sofaer et al., 2019). In this study, both the AUCROC and AUCPR are underestimated by PO approach in most cases. Take the classification of urban as an example, the AUCROC and AUCPR values by GLM trained with a sample size of 200 are 0.9529 and 0.8303 for the PA approach, but the AUC values become 0.8474 and 0.4148 for the PO approach. Please note that the ROC curve of a null model is a straight line connecting the points (0, 0) and (1, 1) in the ROC space, showing that *TPR* is equal to *FPR*. By contrast, the ROC curve of a trained model is usually higher than that of a null model, showing that *TPR* is larger than *FPR*. In other words, *m*_2_/(*m*_2_ + *m*_5_) is larger than *m*_3_/(*m*_3_ + *m*_6_) for a trained model according to Table 2. Consequently, (*m*_2_ + *m*_3_)/(*m*_2_ + *m*_5_ + *m*_3_ + *m*_6_) is larger than *m*_3_/(*m*_3_ + *m*_6_), that is, *FPR*′ of PO approach is larger than *FPR* of PA approach. Meanwhile, *TPR*′ (equivalent to *r*′) of PO approach is equal to *TPR* (equivalent to *r*) of PA approach. As a result, the ROC curve and AUCROC of a trained model by PO approach are usually lower than that by PA approach. Meanwhile, we can infer that *p*′ = *p*/[*p* + (1 − *c*)/*c*] according to Equation (9). In this study, *c* ranges from 0.2 to 0.8 and *p* ranges from zero to one, so *p* is larger than *p*′ in most cases, which is the reason why the PR curve and AUCPR of a trained model by PO approach are also lower than that by PA approach in our experiment.

Unlike the PO approach that treats all of the background data as absence data, the PB approach acknowledges that background data contain both presence and absence data, and it infers the true performance of a model based on a constant *c*. According to our experimental results, the PB approach is effective in calibrating the ROC/PR curves given that the true value of *c* is known. The curves and AUC values by PB approach are very similar to that by PA approach. In reality, however, the true value of *c* is usually unknown and hence it has to be estimated. According to Equation (9), an overestimate of *c* will result in an underestimate of *p*. Because *p* is negatively related to commission error whereas *FPR* is positively related to commission error, an underestimate of *p* will result in an overestimate of *FPR*. These are the reasons why the ROC/PR curves and AUC values are underestimated when *c* is overestimated. Since the largest absolute value of relative error of *c* is 6.56% in this study, we tested the sensitivity of the calibrated curves by PB to *c* with additive relative errors of ±10%, and the results show that the curves and AUC values by PB with the largest relative error of *c* are still better than that by PO approach. Previous research has indicated that the ROC curve and AUCROC value may be inflated when a large number of *TN* data exist in a confusion matrix (Lobo et al., 2008). By contrast, the PR curve does not consider *TN* data in a confusion matrix so it is more robust to geographic extent and species prevalence, but it has a more variable shape than the ROC curve especially at the positions with low values of *r* (Boyd et al., 2012; Sofaer et al., 2019). Consistently, we can observe that the AUCROC values are larger than the AUCPR values, and the ROC curves are generally more stable than the PR curves. In the sensitivity analysis of *c*, the variation of the ROC curve is smaller than that of the PR curve probably because the effect of *c* is offset by a large number of *TN* data.

In this study, the ratio of presence to background data in the test set is empirically set as 1:5. According to our test, changing this ratio value does not affect the PB‐based ROC/PR plots because the derivation of *c* is unrelated to the ratio (see Figure 5). When we fix the number of presence data but change the number of background data in the test set, the values of *TPR*′ (or *r*′) and *FPR*′ are unaffected; however, the value of *p*′ will become lower with a larger number of background data according to Equation (12). Therefore, the AUCROC value by PO approach is also unaffected by the ratio, but the AUCPR value by PO approach is greatly affected by the ratio. In practice, it is reasonable to use a larger number of background data than the presence data since the background data are samples that represent both classes (presence and absence), but we do not recommend using a huge number of background data which will produce an extremely unbalanced test set.

The proposed PB method to calibrate the ROC/PR curves is based on the work of Li and Guo (2013). The omission error is related to *r* whereas the commission error is related to *p*, both of which are quantified in the ROC/PR plots. The relationship between *r* and *r*′ and the relationship between *p* and *p*′ derived in Li and Guo (2013) are used to reconstruct the true ROC/PR curves from presence and background data. The key information of this PB method is the constant *c* or species prevalence. Although true species prevalence is regarded as unidentifiable without absence data, an estimation of prevalence is helpful and possible under certain conditions (Hastie & Fithian, 2013; Li et al., 2011; Phillips & Elith, 2013; Royle et al., 2012). Please note that *r* is equal to *r*′, and the relationship between *p* and *p*′ is similar to the relationship between probability of species occurrence and a naive model fitted from presence‐background data. Let *f* = *P*(*y* = 1|*x*) denote the probability of species occurrence and *f*′ = *P*(*s* = 1|*x*) denote a naive model. We have *f* = *f*′/*c* and *p* = *p*′/*c* in the single‐training‐set scenario, or *f* = (1 − *c*)/*c* × *f*′/(1 − *f*′) and *p* = (1 − *c*)/*c* × *p*′/(1 − *p*′) in the case–control scenario (Elkan & Noto, 2008; Li et al., 2011). Therefore, an estimation of *c* (or prevalence) not only enables us to model the probability of species occurrence, but also helps us to assess the model performance without requiring labeled absence data.

There are several ways to estimate the constant *c* (or prevalence). Li et al. (2011) proved that the average predicted values of *f*′ at prototypical presence sites where the habitats are maximally suitable for a species can be used to estimate *c*, but this approach may lead to an underestimate of *c* because the probability of species occurrence at a selected prototypical presence site may be smaller than one. Royle et al. (2012) proposed the MAXLIKE that can be used to infer prevalence, but the linear logistic model assumption may be violated in reality (Guillera‐Arroita et al., 2015; Hastie & Fithian, 2013; Phillips & Elith, 2013). Li and Guo (2013) showed that thresholding a naive model based on maximizing the measure *F*_pb_ on a validation set can also estimate prevalence, but Liu et al. (2016) and Leroy et al. (2018) argued that it is difficult to estimate prevalence using threshold‐based approach. In this study, we propose to estimate *c* from the ROC/PR plots based on the fact that a model of good discrimination ability can produce the maximum value of *p* (or minimum value of *FPR*) with a high value of threshold. In other words, we can adjust the PR curve so that its highest point reaches the maximum value of one (equivalent to adjusting the ROC curve so that its most left point reaches the minimum value of zero), and the relationship between *p* and *p*′ yields an estimate of *c*. Because there could be multiple threshold values that can produce maximum value of *p*, we use multiple points rather than the highest point in the PR curve to obtain a more robust estimate of *c*, for example, the higher threshold values between 90th and 99th percentiles. In our experiment, this percentile range consistently produces high accuracies of *c* for different species, and it can be adjusted in other situations. Meanwhile, the discrimination ability of a model can affect the accuracy of *c*. The largest relative error of *c* is observed for the classification of tree because the model cannot perform well in discriminating tree from other land types, with the lowest value of AUCPR compared with other species. This is because a model with lower AUC value will have the lower probability to correctly rank the predicted probabilities. As a result, the number of points that are suitable to estimate *c* in the ROC/PR plots becomes smaller as the discrimination ability (measured by AUC) of a model decreases, so the default percentile range (i.e., 90th to 99th percentiles) might not be appropriate. According to our test, a model with a low AUCROC value like 0.6 (slightly better than a null model whose AUCROC value is 0.5) is still able to estimate *c*, but it is necessary to carefully select the optimal points in the curves where *FPR* is close to zero or *p* is close to one. Intuitively, we can observe that the ROC curve starts from the point (0, 0) where *FPR* is zero, and a trained model which is better than a null model will shift the curve upward, so it is possible to find points where *FPR* is equal or close to zero to estimate *c*, excluding the point (0, 0) where *TPR* is also zero. If possible, users can also derive *c* (or prevalence) from other sources such as limited presence–absence surveys or expert knowledge (Phillips & Elith, 2013). However, the uncertainty of *c* is almost inevitable no matter it is derived from models or surveys.

In this study, we only focus on calibrating the ROC/PR curves from presence and background data, but model evaluation may involve multiple aspects and multiple measures. The strengths and drawbacks of ROC/PR plots have been well investigated in the literature (Boyd et al., 2012; Davis & Goadrich, 2006; Fielding & Bell, 1997; Lobo et al., 2008; Sofaer et al., 2019). For example, the current ROC/PR plots have been criticized to equally weigh the commission and omission errors, but these two types of errors may not be of the same importance (Lobo et al., 2008; Peterson et al., 2008). In spite of the limitations of ROC/PR plots, the proposed method can be used as a complement to other model assessment methods. For example, the presence‐only calibration (POC) plot by Phillips and Elith (2010) can be used to measure the ability of calibration for continuous outputs. The AVI and CVI in Hirzel et al. (2006), *F*_pb_ and *F*_cpb_ in Li and Guo (2013), and TSS in Liu et al. (2013) can assess the accuracy of binary outputs without requiring absence data. Other methods such as Boyce index and the compositional and multinomial procedure can also be considered to quantify model performance from different aspects when absence data are not available (Boyce et al., 2002; Ottaviani et al., 2004).

Similar to the proposed PB‐based ROC/PR plots, both the POC plot and *F*_cpb_ also require additional information on the constant *c* (or species prevalence) (Li & Guo, 2013; Phillips & Elith, 2010). Actually, the four quadrants of a confusion matrix can be fully determined from presence and background data if *c* is known, and all of the accuracy measures derived from a confusion matrix can be calculated, which should be investigated in future research. Although different approaches to estimate *c* still have their limitations, such attempts are necessary. In practice, users can consider applying multiple approaches to reduce the uncertainty of *c*. Since *c* = *n*_1_/[*n*_1_ + *n*_0_ × *P*(*y* = 1)] in the case–control scenario, *n*_1_/(*n*_1_ + *n*_0_) ≤ *c* ≤ 1 because 0 ≤ *P*(*y* = 1) ≤ 1. If we can provide a rough estimate of prevalence such as from survey or expert knowledge, then the range of *c* can be refined. Meanwhile, the estimator derived from prototypical presences in Li et al. (2011) usually underestimates *c*, so it can be used as the lower bound of *c*.

In this study, the ROC/PR curves plotted from PA approach are used to benchmark the curves from PO and PB approaches, so a test set with presence–absence data is required. Because we do not have real species datasets with reliable absence data, we only tested the proposed method using virtual species datasets. In addition, we used a real aerial photograph since model evaluation of binary classification in remote sensing is mathematically similar to model evaluation in SDM, and both presence and absence data can be obtained through manual interpretation of the aerial photography. However, there are still some differences between remote sensing classification and SDM due to the complicated biological processes such as biotic interactions (Warren et al., 2020). Therefore, the proposed PB method should be further investigated using real species datasets in the future when reliable species absence data are available.

## 5 CONCLUSION

In this study, we propose a new PB approach to plot the ROC/PR curves from presence‐background data with additional information of a constant *c* (or species prevalence). The accuracy measures *r* and *p* derived from presence–absence data can be connected to *r*′ and *p*′ derived from presence‐background data through the constant *c*, which enables reconstructing the true ROC/PR curves from presence‐background data. Meanwhile, *c* can be estimated from the ROC/PR plots under the condition that a model of good discrimination ability exists. Our experimental results demonstrate that the proposed PB approach is effective both in plotting the ROC/PR curves and estimating *c* from presence‐background data in the case–control sampling scenario.

## CONFLICT OF INTEREST

The authors declare no conflict of interest.

## AUTHOR CONTRIBUTIONS

**Wenkai Li:** Conceptualization (lead); methodology (lead); writing‐original draft (lead); writing‐review & editing (lead). **Qinghua Guo:** Conceptualization (supporting); methodology (supporting); writing‐original draft (supporting); writing‐review & editing (supporting).

## Data Availability

The data used to simulate virtual species are available at WorldClim database (https://worldclim.org/). The simulated species data are available in Dryad (https://doi.org/10.5061/dryad.b8gtht7cp).
